# Long‐term outcomes in patients with postural orthostatic tachycardia syndrome with an average follow‐up of over 20 years

**DOI:** 10.1111/joim.70104

**Published:** 2026-05-19

**Authors:** Kate M. Bourne, Alfredo Gamboa, Bonnie Black, Juliette Hall, Italo Biaggioni, Cyndya A. Shibao, André Diedrich, Amanda Peltier, Giris Jacob, Luis Okamoto, Robert S. Sheldon, Satish R. Raj

**Affiliations:** ^1^ Department of Cardiac Sciences Libin Cardiovascular Institute Cumming School of Medicine University of Calgary Calgary Alberta Canada; ^2^ Vanderbilt Autonomic Dysfunction Center Division of Clinical Pharmacology Department of Medicine Vanderbilt University Medical Center Nashville Tennessee USA; ^3^ J. Recanati Autonomic Dysfunction Center Tel Aviv Sourasky Medical Center Tel Aviv Israel

**Keywords:** outcomes, postural orthostatic tachycardia syndrome

## Abstract

**Background:**

Postural orthostatic tachycardia syndrome (POTS) is a chronic form of orthostatic intolerance that primarily affects female patients. There are scarce data evaluating the long‐term outcomes in POTS.

**Objectives:**

This study sought to evaluate the long‐term impacts of POTS over multiple decades in adult patients.

**Methods:**

Past research participants at the VUMC Autonomic Dysfunction Center Research Unit (symptomatic ≥10 years) were recruited to participate in the study. A custom survey was administered at one time point. Participants were grouped as IMPROVED or NOT IMPROVED based on symptom course over time. Continuous data are reported as median (25th, 75th).

**Results:**

Patients with POTS (*n* = 44; 98% female) were included in the analysis (62% response rate). Patient age at the time of survey was 48 (38, 54) years, with 23 (15, 27) years from POTS symptom onset, and 17 (12, 24) years from POTS diagnosis. Since diagnosis, symptoms completely resolved in 2%, improved in 46%, worsened in 25%, were unchanged in 11%, and demonstrated a variable symptom course in 16%. Patients who were NOT IMPROVED were more likely than those IMPROVED to have neuropathy, gastroparesis, and overactive bladder symptoms.

**Conclusions:**

In a cohort of adult patients with POTS who received care at a national referral center for autonomic disorders, almost half reported their POTS symptoms as improved 10 or more years after symptom onset. Most patients with POTS experienced ongoing symptoms for many years after diagnosis.

## Graphical abstract

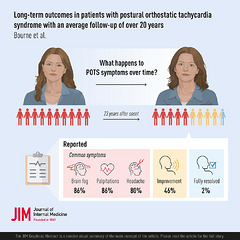

**Abbreviations**
COMPASS‐31: composite autonomic symptom score‐31
EDS: Ehlers–Danlos syndrome
HR: heart rate
MCAS: mast cell activation syndrome
OI: orthostatic intolerance
PCP: primary care physician
POTS: postural orthostatic tachycardia syndrome
REDCap: research electronic data capture
VOSS: Vanderbilt Orthostatic Symptom Score
VUMC: Vanderbilt University Medical Center

## Introduction

Postural orthostatic tachycardia syndrome (POTS) is a chronic form of orthostatic intolerance (OI) that primarily impacts female patients [1]. The diagnostic criteria for POTS require a sustained heart rate increase of ≥30 bpm within 10 min of upright posture, without orthostatic hypotension (>20/10 mmHg), and in association with chronic orthostatic symptoms [2, 3, 4]. Patients with POTS experience debilitating symptoms that significant impacts quality of life [5], and many are unable to work due to the severity of their illness, resulting in substantial economic loss [6].

Many patients with POTS ask about their long‐term prognosis. There are only a few studies addressing outcomes in POTS, and these report on primarily short‐term outcomes and mainly in young patients [7, 8, 9, 10, 11, 12, 13, 14]. Among practitioners, there is a misconception that POTS will spontaneously resolve over time, although there is no evidence to support these claims. The long‐term outcomes in adult POTS patients are not known. We sought to address this important gap in the literature by evaluating the long‐term outcomes over several decades in adult POTS patients using an online survey. This survey was administered retrospectively to a group of patients with POTS who were previously evaluated and managed at the Autonomic Dysfunction Center Research Unit at Vanderbilt University Medical Center (VUMC). We hypothesized that most patients would experience some improvement, but not a full resolution, of their POTS symptoms over several years.

As it is unclear why some patients with POTS may experience symptom improvement whereas other patients experience persistent and potentially worsening symptoms for several years, we also sought to further understand the long‐term symptom course of POTS by evaluating differences between patients with worsening or no change in POTS symptoms compared to patients with improved POTS symptoms.

## Methods

### Survey design

A retrospective cohort study was designed jointly by VUMC and the University of Calgary. The first aim of the survey was to evaluate the long‐term outcomes of POTS, including symptom course, treatment history, and quality of life, as well as the educational, employment, and social impacts of living with POTS over the long term (Aim 1). The second aim was to compare participants who reported an improvement in POTS symptoms to those who reported no change or worsening symptoms, to attempt to identify factors influencing improvement over time (Aim 2). The survey was administered at one time point and included a combination of standardized questionnaires (Composite Autonomic Symptom Score‐31 [COMPASS‐31], Autonomic Symptom Scale [15], RAND‐36 Health Related Quality of Life [16], and symptoms from the Vanderbilt Orthostatic Symptom Score [VOSS] [17]), as well as specific questions surrounding diagnosis, symptoms, treatments, and the multifactorial impacts of POTS (Supporting Information section). Common comorbidities were selected based on findings from a large survey of patients with POTS [18], as well as the clinical experiences of the research team. The survey was delivered online through the secure Research Electronic Data Capture (REDCap) platform housed at Vanderbilt University [19]. This study received ethical approval from Vanderbilt University (IRB#210224) and the University of Calgary (REB22‐1707).

### Participants

#### Aim 1

Participant recruitment took place at VUMC. All participants had a physician diagnosis of POTS and were previously evaluated at the VUMC Autonomic Dysfunction Center Research Unit. All participants were from the United States and were adults at the time of the survey (range: 30–67 years of age). Potential participants with POTS symptom onset >10 years prior were contacted by a VUMC research nurse, and those who agreed to participate were sent a unique REDCap Survey link. The 10‐year time frame was selected to evaluate the long‐term experience. Most other literature uses shorter time horizons. Participants provided informed consent through an electronic informed consent form on the REDCap platform. Participants were required to use a device with internet access to complete the informed consent form and survey. To increase survey response rates, potential participants who agreed to participate, but had not yet completed the survey, received three additional reminder emails through REDCap in 7‐day intervals, in addition to the initial survey email. After the third reminder email, attempts to contact the potential participant were ceased.

#### Aim 2

Only participants who indicated their symptoms as “fully recovered,” “improved,” “worse,” or “no change” at the time of the survey were included (*n* = 37). Participants who indicated their symptoms as “variable” (*n* = 7) were excluded. Participants who indicated their POTS symptoms were “fully recovered” or “improved” were grouped together as IMPROVED, and participants who indicated their POTS symptoms as “no change” or “worse” were grouped together as NOT IMPROVED.

### Survey data

Survey data were collected between March and September 2021. Data were collected from patient recollection regarding questions related to both the time of diagnosis (or time of first visit at VUMC) and the time of the survey. If a participant did not answer a specific question, then they were excluded from the total number of respondents for that specific question. The total number of POTS‐related comorbidities was calculated by adding together the positive responses for each comorbidity question (out of a total of 10). The total number of POTS medications (out of 14) and non‐pharmacological treatments (out of 6) were calculated in a similar way, by adding together the responses for each individual question. The age of symptom onset and age of diagnosis were calculated by subtracting the year of onset and year of diagnosis from the survey year. Participants were asked if POTS impacted different areas of their lives. If yes, they were then asked to provide a free‐text description. The free‐text descriptions were analyzed and coded as “negative” or “positive” impact. This coding was used to determine the number of participants who experienced negative impacts in each category. Participants were asked why they were not currently seeing a primary care physician (PCP) for POTS. If participants selected “other reason” as an answer, they were asked to provide a free text answer. The free text answers were analyzed and grouped into categories. Participants were grouped into adult‐onset or pediatric‐onset POTS based on the age of POTS symptom onset (<18 years for pediatric‐onset and ≥18 years for adult‐onset), although all patients with POTS were ≥18 years when seen initially at VUMC.

### Statistical analysis

***Aim 1***: Data were exported from REDCap and imported into IBM SPSS Statistics 28 (Armonk, NY) for statistical analysis. Summary statistics were calculated. Categorical variables are presented as the percentage of participants, and continuous variables are presented as median (25th, 75th percentile). Wilcoxon Signed Rank tests were used to compare continuous data between the time of diagnosis and the time of the survey, or the time of visit at VUMC and the time of the survey. ***Aim 2***: Fisher's exact tests were used to compare categorical variables, and Mann–Whitney *U* tests were used to compare continuous variables, between NOT IMPROVED and IMPROVED. A *p* value of <0.05 was considered statistically significant. Bonferroni correction for multiple comparisons was applied to COMPASS‐31 and RAND46 scores as well as symptoms and comorbidities described in Tables S2 and S3.

## Results

### Aim 1: Survey participants

**Fig. 1:**
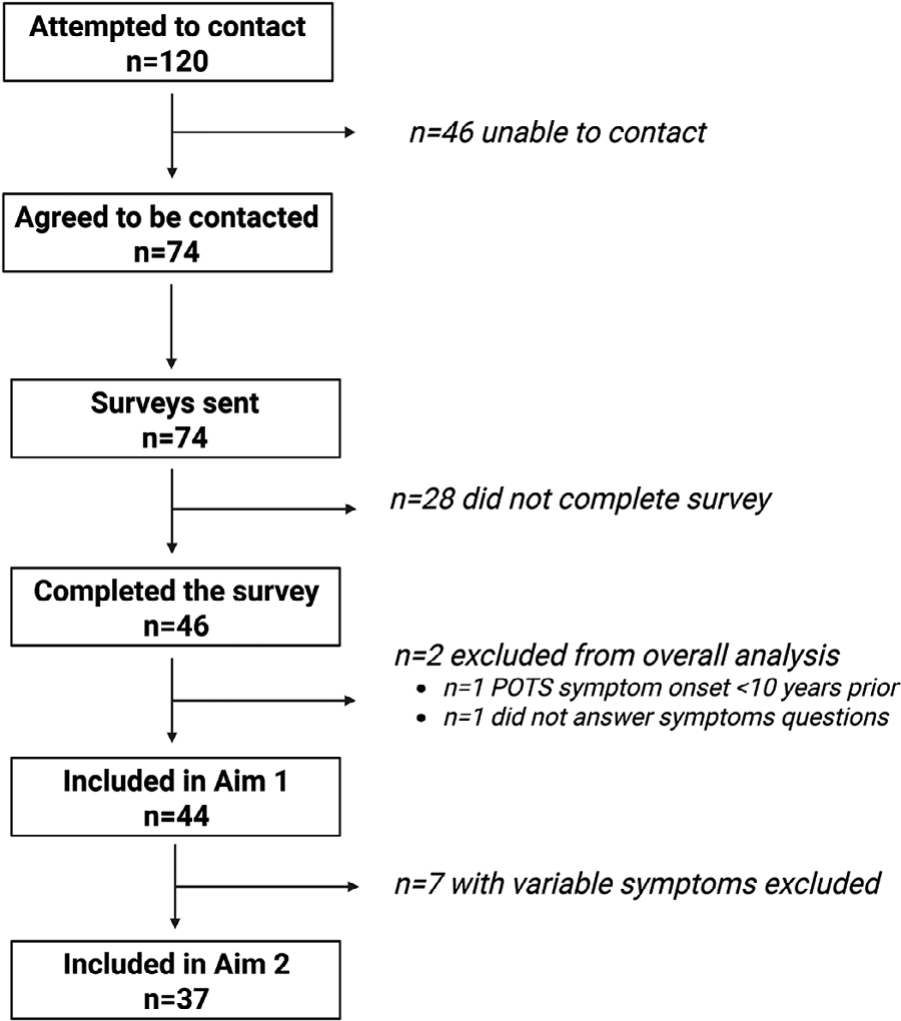
Flow diagram of participant recruitment, enrollment, and inclusion in analyses.

We tried to contact 120 individuals who had initially attended the Autonomic Dysfunction Center Research Unit at VUMC between 1987 and 2014. In total, 74 participants responded to contact, and 46 participants completed the survey (62% response rate) between March and September 2021. Not all participants were still being regularly followed at VUMC. One participant reported POTS symptom onset <10 years prior to the survey date, and one person did not answer the questions regarding POTS symptom changes. They were both excluded from further analysis. Therefore, a total of 44 participants with POTS were included in the analysis (Fig. 1).

### Demographics

Most participants (98%, *n* = 43) were female and identified as women (all participants identified as cisgender; Table 1). All participants identified their race as White. The median age of participants at the time of POTS symptom onset was 26 (17, 35) years, at the time of diagnosis was 31 (26, 38) years, and at the time of survey was 48 (38, 54) years. The median duration of time since POTS symptom onset was 23 (15, 27) years, and the duration of time since POTS diagnosis was 17 (12, 24) years.

**Table 1:** Demographics.

Demographic	*n* (%)
Sex	
Female	43 (98)
Male	1 (2)
Gender	
Woman	43 (98)
Man	1 (2)
Race	
White	44 (100)
Ethnicity^a^	
Hispanic	2 (5)
Non‐Hispanic	41 (95)

	Median (25th, 75th percentile)
Age of POTS symptom onset (years)	26 (17, 35)
Age of POTS diagnosis (years)	31 (26, 38)
Survey age (years)	48 (38, 54)
Duration since POTS symptom onset (years)	23 (15, 27)
Duration since diagnosis (years)	17 (12, 24)
Doctors seen before diagnosis	5 (3, 9)
Diagnostic delay (years)	2 (1, 8)

Abbreviations: POTS, postural orthostatic tachycardia syndrome.

^a^ Out of 43 participants.

### Course of POTS symptoms over time

**Fig. 2:**
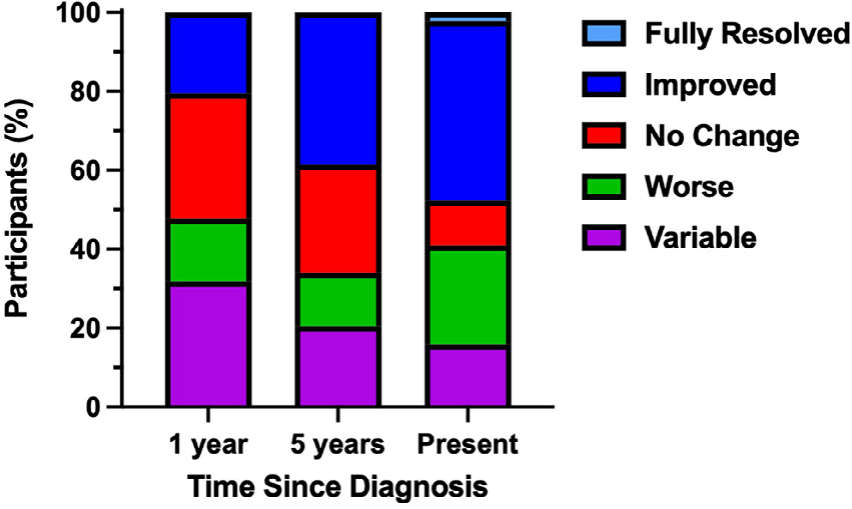
Change in postural orthostatic tachycardia syndrome (POTS) symptoms up to 1 year after diagnosis, 5 years after diagnosis, and overall, compared to time of diagnosis.

Most participants (98%, *n* = 43) reported that they continued to have POTS symptoms because they were diagnosed with POTS. Up to 1 year after diagnosis, 21% (*n* = 9) of participants reported that their POTS symptoms had improved, and 16% (*n* = 7) reported that their symptoms had worsened, compared to the time of diagnosis (Fig. 2). At 5 years after diagnosis, 39% (*n* = 17) of participants reported that their POTS symptoms had improved, and 14% (*n* = 6) reported that their symptoms had worsened, compared to the time of diagnosis. Just under half of participants (48%) reported a stable change in symptoms (from diagnosis) at 1 and 5 years.

From diagnosis to the time of the survey, 2% (*n* = 1) of participants reported that their POTS symptoms had completely resolved, 46% (*n* = 20) reported that their symptoms had improved, and 25% (*n* = 11) reported that their symptoms had worsened. Half of participants reported a stable change in symptoms from 5 years after diagnosis to the time of the survey. The proportions of participants with no change in symptoms, or variable symptoms, decreased over time (Fig. 2). Excluding participants with variable symptoms at the time of the survey (*n* = 7), just over half of participants (57%, *n* = 21/37) reported that their POTS symptoms had improved, and 43% (*n* = 16/37) reported that their POTS symptoms were unchanged or had worsened at the time of the survey, compared to the time of diagnosis.

### Pediatric versus adult‐onset POTS

Most participants (73%, *n* = 32) reported POTS symptom onset at age 18 years or older. There was no significant difference in the proportion of participants with improved symptoms at the time of the survey in the pediatric‐onset compared to adult‐onset groups (42% vs. 50%, *p* = 0.7).

### POTS comorbidities

Common comorbidities included headaches (68%), irritable bowel syndrome (48%), neuropathies (39%), and chronic fatigue syndrome/myalgic encephalomyelitis (36%; Table 2). Of those with neuropathy, 47% reported small fiber neuropathy, 35% reported peripheral neuropathy (not specified as small fiber), and 18% were unsure of the type of neuropathy. At least one of the comorbidities in Table 2 was reported by 86% of participants (*n* = 38/44), and the median number of comorbidities was 3 (1, 5).

**Table 2:** Proportion of participants with comorbidities commonly associated with postural orthostatic tachycardia syndrome (POTS).

Comorbidity	*n* (%)
Headache	30 (68)
Irritable bowel syndrome	21 (49)^a^
Neuropathy	17 (40)^a^
Chronic fatigue syndrome/myalgic encephalomyelitis	16 (36)
Overactive bladder symptoms	12 (27)
Autoimmune disease	12 (27)
Gastroparesis	8 (18)
Hypermobile Ehlers–Danlos syndrome	8 (18)
Mast cell activation syndrome	4 (9)
Chiari malformation	3 (7)
At least one of the above	38 (86)

^a^ Out of 43 participants.

### Current symptoms

**Fig. 3:**
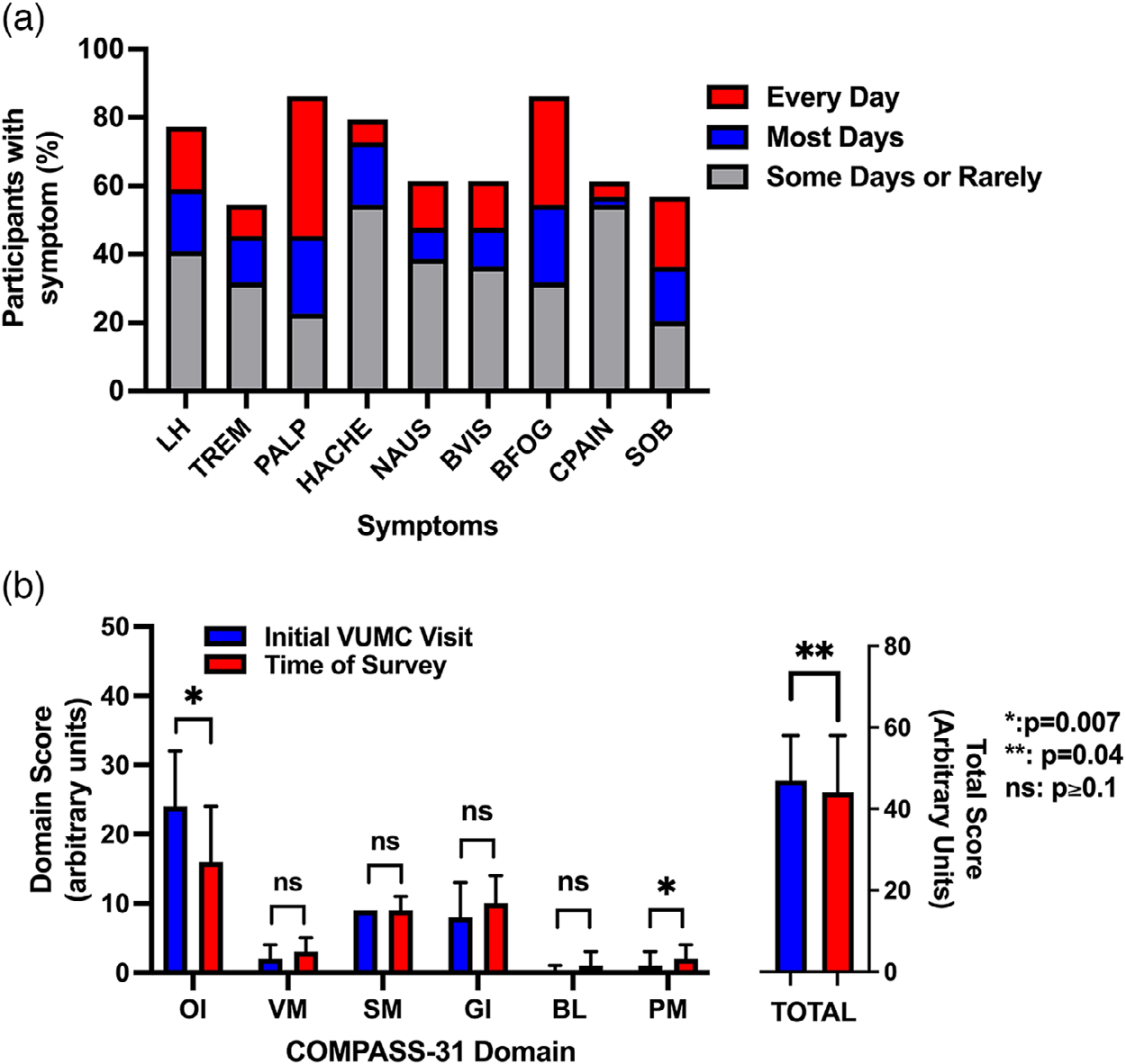
(a) Symptoms of orthostatic intolerance at the time of the survey. The overall proportion of participants with the symptom is shown, along with the proportions of participants who experience each symptom every day (7 days/week), most days (4+ days/week), and some days or rarely (up to 2 days/week). (b) COMPASS‐31 Autonomic Symptom Score at the time of the survey compared to the time of the first visit at VUMC. A higher number indicates a greater symptom burden. Data are presented as median and interquartile range. BFOG, brain fog; BL, bladder; BVIS, blurry vision; CPAIN, chest pain; GI, gastrointestinal; HACHE, headache; LH, lightheadedness; NAUS, nausea; OI, orthostatic intolerance; PALP, palpitation; PM, pupillomotor; SM, secretomotor; SOB, shortness of breath; TOT, total score; VM, vasomotor.

Participants were asked to report upright VOSS symptoms at the time of the survey: brain fog (86%, *n* = 38), rapid heartbeat (86%, *n* = 38), headache (80%, *n* = 35), lightheadedness (77%, *n* = 34), blurry vision (63%, *n* = 27), chest pain (63%, *n* = 27), nausea (62%, *n* = 27), shortness of breath (57%, *n* = 25), and tremulousness (55%, *n* = 24, Fig. 3a). One‐quarter (25%, *n* = 11) of participants reported all 9 of these symptoms. More than half of participants with the following symptoms reported experiencing them every day, or most days: rapid heartbeat (74%, *n* = 28), brain fog (63%, *n* = 24), and shortness of breath (64%, *n* = 16). Almost half of participants with lightheadedness reported experiencing this every day or most days (47%, *n* = 16).

### General health visual analogue scale

On a scale of 0–100 (100 is best health), participants rated their overall health as better at the time of the survey (50 [35, 75] arbitrary units (au)) compared to the time of their first appointment at VUMC (36 [20, 50] au; *p* < 0.001).

### COMPASS‐31 autonomic symptom burden

Participants reported reduced symptom burden (lower COMPASS‐31 scores) at the time of the survey compared to when first seen at VUMC in the OI domain score, as well as the total COMPASS‐31 score (Fig. 3b). Conversely, autonomic symptom burden was higher (higher COMPASS‐31 scores) at present time compared to when first seen at VUMC in the pupillomotor domain. Vasomotor, secretomotor, gastrointestinal, and bladder symptom domains did not significantly change over time (Fig. 3b).

### Pharmacological and non‐pharmacological treatments

Current use of at least one POTS medication was reported by 66% of participants (median number of POTS medications: 1 [0,2]; Table 3), and 27% of participants reported using one or more POTS medications in the past. Over half of participants were taking a beta‐blocker at the time of the survey (57%). Most participants (82%) were using at least one non‐pharmacological treatment at the time of the survey (median 3 [1,4]), including increased salt (66%) and fluids (75%) in their diet (Table 4). All participants had tried one or more non‐pharmacological treatments at some point in time for POTS. Overall, at the time of the survey, 55% (*n* = 24) of participants were using both non‐pharmacological treatments and medications for their POTS, 11% (*n* = 5) were using POTS medications but no non‐pharmacological treatment, 27% (*n* = 12) were using non‐pharmacological treatment but no POTS medications, and 7% (*n* = 3) were not using either POTS medication or non‐pharmacological treatment.

**Table 3:** Proportion of participants currently taking, or who have taken, medications for postural orthostatic tachycardia syndrome (POTS), and symptom change in response to medication.

Medication	Currently taking *n* (%)	Have taken *n* (%)	Improved a lot *n* (%)	Improved a little *n* (%)	No change or worse *n* (%)
Beta‐blocker	25 (57)	14 (32)	16 (41)	12 (31)	9 (23)
Midodrine	3 (7)	19 (43)	1 (5)	8 (36)	11 (50)
Fludrocortisone	5 (11)	29 (66)	5 (15)	9 (27)	17 (50)
Ivabradine^a^	1 (2)	3 (7)	1 (25)	1 (25)	2 (50)
Pyridostigmine	1 (2)	7 (16)	0 (0)	0 (0)	7 (88)
Methyldopa	1 (2)	5 (11)	2 (33)	0 (0)	3 (50)
Clonidine	3 (7)	5 (11)	1 (11)	3 (38)	4 (50)
Desmopressin	0 (0)	1 (2)	0	1 (2)	0 (0)
Modafinil	2 (5)	6 (14)	3 (38)	1 (13)	4 (50)
Armodafinil	0 (0)	1 (2)	0 (0)	0 (0)	1 (100)
LDN	1 (2)	0 (0)	0 (0)	0 (0)	1 (100)
EPO	1 (2)	3 (7)	1 (25)	1 (25)	2 (50)
IVIG	1 (2)	2 (5)	1 (33)	1 (33)	1 (33)
Other for POTS	8 (18)	3 (7)	6 (75)	1 (13)	1 (13)

Abbreviations: EPO, erythropoietin; IVIG, intravenous immunoglobulin; LDN: low‐dose naltrexone; POTS, postural orthostatic tachycardia syndrome.

^a^ Out of 43 participants.

**Table 4:** Proportion of participants currently using, or who have used, non‐pharmacological treatments for postural orthostatic tachycardia syndrome (POTS), and symptom change in response to the treatment.

Treatment	Currently using *n* (%)	Have tried *n* (%)	Improved a lot *n* (%)	Improved a little *n* (%)	No change or worse *n* (%)
Increased dietary salt	29 (66)	14 (32)	10 (23)	20 (47)	8 (19)
Increased oral fluids	33 (75)	9 (21)	17 (41)	22 (52)	1 (2)
Electrolyte supplements	12 (27)	14 (32)	5 (19)	8 (31)	11 (42)
Compression garments	10 (23)	26 (59)	3 (8)	20 (56)	9 (25)
Exercise program	16 (36)	16 (36)	10 (31)	9 (28)	10 (31)
IV Saline—emergency use	6 (14)	14 (32)	10 (50)	8 (40)	1 (5)

### Physicians

At the time of the survey, 59% (*n* = 25) of participants were seeing a physician for POTS. About one third (32%, *n* = 8) were seeing both a PCP and a specialist, 32% (*n* = 8) were seeing a PCP only, and 36% (*n* = 9) were seeing a specialist only. Participants not seeing a PCP for POTS were asked for the reasons as to why: 38% (*n* = 10) said their symptoms were well managed, 31% (*n* = 8) did not have or could not find a PCP who understands and is comfortable treating POTS, 23% (*n* = 6) saw their specialist instead, 4% (*n* = 1) reported insurance concerns, and 4% (*n* = 1) indicated the doctor was too far away from them. Further information about physicians can be found in Table S1.

### Female participants

Most participants were female (98%, *n* = 43). Over half of participants were premenopausal (60%, *n* = 25), and 41% (*n* = 17) reported they were postmenopausal at the time of the survey. More information about female participants can be found in Table S1.

### Impacts of POTS

**Fig. 4:**
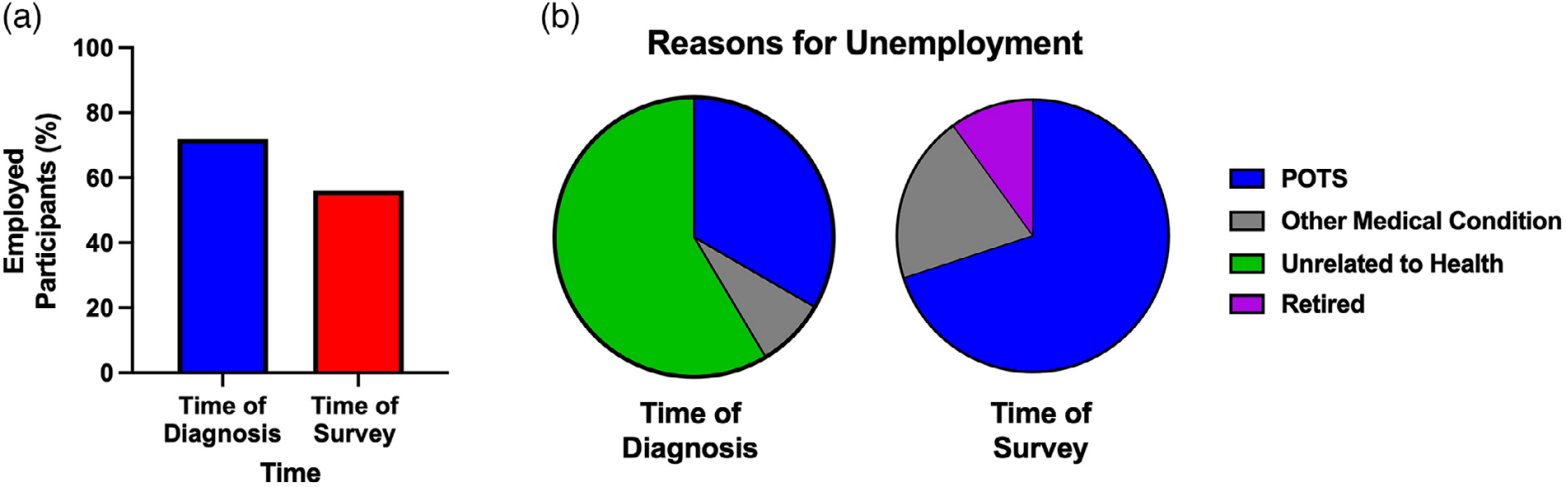
(a) Percentage of participants employed at the time of postural orthostatic tachycardia syndrome (POTS) diagnosis and at the time of the survey. (b) Reasons for unemployment at the time of POTS diagnosis (left) and the time of the survey (right).

Overall, 68% (*n* = 30) of participants reported that POTS changed their career over time. At the time of POTS diagnosis, 72% (*n* = 31) of participants were employed (81% full‐time; Fig. 4a). Of participants who were unemployed (*n* = 12), 33% (*n* = 4) reported this was due to POTS, 8% (*n* = 1) reported this was due to another medical condition, and 58% (*n* = 7) reported that it was for another reason unrelated to health (Fig. 4b). At the time of the survey, 55% (*n* = 24) of participants were employed (75% full‐time). Of unemployed participants, 70% (*n* = 14) were unable to work due to POTS, 20% (*n* = 4) were unable to work due to another medical condition, and 10% (*n* = 2) had retired. This is equivalent to 32% of the overall cohort who were unable to work due to POTS. Further information about impacts, employment, and disability benefits are included in Table S1.

### Aim 2: NOT IMPROVED compared to IMPROVED

#### Onset, diagnosis, and symptoms

There were no significant differences in age of POTS symptom onset, length of diagnostic delay, and time since diagnosis on NOT IMPROVED (43% *n* = 16) compared to IMPROVED (57% *n* = 21; Fig. S1). More NOT IMPROVED versus IMPROVED reported tremulousness (81% vs. 24%, *p* = 0.02), nausea (94% vs. 29%, *p* = 0.009), blurry vision (94% vs. 40%, *p* = 0.009), shortness of breath (81% vs. 24%, *p* = 0.009), and all nine orthostatic symptoms (56% vs. 0%, *p *< 0.001; Table S2).

#### Comorbidities

More NOT IMPROVED versus IMPROVED participants reported gastroparesis (44% vs. 0%, *p* = 0.01) and neuropathy (67% vs. 10%, *p* = 0.01; Table S3). All but one NOT IMPROVED participant (94%) had one or more of gastroparesis, neuropathy and overactive bladder symptoms, compared to a minority of IMPROVED (19%, *p* < 0.001). The median number of comorbidities (out of the 10 listed in Table S3) was larger in NOT IMPROVED (5 [3, 6] comorbidities) compared to IMPROVED (1 [1, 2] comorbidities, *p* < 0.001).

#### COMPASS‐31 scores

There were no differences in COMPASS‐31 domain scores or total score between NOT IMPROVED and IMPROVED at the time of first visit at VUMC, including in the OI domain (22 [20, 27] vs. 24 [22, 28], *p* = 0.7; Table S3). Current COMPASS‐31 OI domain scores were higher in NOT IMPROVED compared to IMPROVED (20 [20, 24] vs. 12 [8, 16], *p* = 0.007). Vasomotor, gastrointestinal, bladder and pupillomotor domain scores, and the total COMPASS‐31 score (49 [40, 52] vs. 36 [26, 43], *p* < 0.001) were also higher in NOT IMPROVED versus IMPROVED. Vasomotor, gastrointestinal, bladder, pupillomotor, and secretomotor domain scores were not significantly different. NOT IMPROVED had smaller changes in OI domain scores and total scores compared to IMPROVED (Table S4).

#### RAND36 scores

**Fig. 5:**
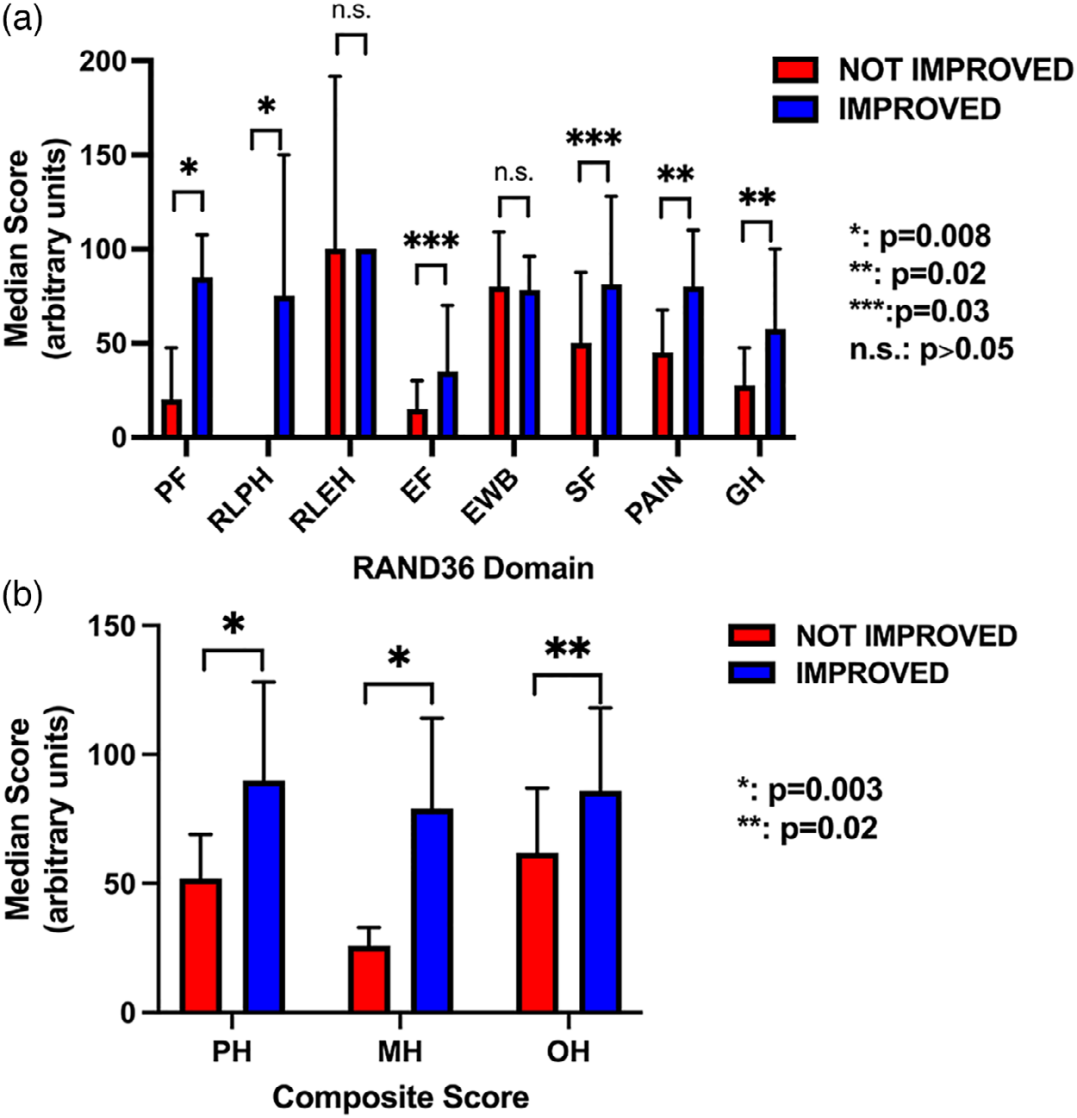
(a) RAND36 health‐related quality of life domain scores in NOT IMPROVED compared to IMPROVED participants. (b) RAND36 health‐related quality of life composite scores in NOT IMPROVED compared to IMPROVED participants. PH: physical health related quality of life composite score, MH: mental health related quality of life composite score, overall health‐related quality of life composite score. EF, energy and fatigue; EWB, emotional well‐being; GH, general health; PAIN, bodily pain; PF, physical functioning; RLEH, role limitations due to emotional health; RLPH, role limitations due to physical health; SF, social functioning.

Current RAND36 Health Related Quality of Life scores were lower (worse quality of life) in NOT IMPROVED, compared to IMPROVED, in the physical functioning, role limitations due to physical health, energy/fatigue, social functioning, pain, and general health domains (Fig. 5a). Role limitations due to emotional health and emotional well‐being were not different. Physical health, mental health, and overall health composite scores were all lower in NOT IMPROVED, compared to IMPROVED (Fig. 5b).

## Discussion

We describe a cohort of adult POTS patients with a median duration of POTS symptoms of over 20 years. Despite receiving care at a specialized autonomic center, all but one participant continued to experience POTS symptoms for years after initial onset and diagnosis. Although patients reported a trend towards improvement of symptoms over time, a complete resolution of symptoms was virtually nonexistent in this cohort of patients. These findings refute the misconception that POTS simply “goes away” or that most patients “grow out” of POTS.

The median age of participants (48 years) was considerably older than reported in the existing literature that primarily focuses on pediatric‐onset POTS and younger cohorts [7, 8, 9, 10, 11, 12, 13]. The median time from symptom onset to survey of 23 years and time from diagnosis to survey of 17 years were also much longer than any of the existing published literature. This helps to paint a more accurate picture of what a person with POTS might experience decades after POTS onset. Additionally, most participants in the survey had adult‐onset POTS, more accurately describing long‐term outcomes in patients who were adult age at the time of onset.

### Symptom burden over time

Over time, the proportions of participants with improved or worsened POTS symptoms increased, and the proportions with no change or variable symptoms decreased. Much of the symptom improvement occurred within the first 5 years after diagnosis, rather than after that time. Symptoms of OI decreased by the time of the survey, whereas other autonomic symptoms like gastrointestinal symptoms increased. In published pediatric and young adult studies, symptom improvement is reported in most participants, with percentage improved reported as upward of 80 of patients [7, 9, 10, 11, 13], considerably higher than our current findings. In a recent long‐term outcomes survey of pediatric patients, however, 1% of participants were asymptomatic in the month prior to the follow‐up survey [14].

It is possible that the mechanisms driving pediatric‐onset POTS could be different than the mechanisms of adult‐onset POTS. In our study, we did not find a significant difference in the percentage of improved participants between pediatric and adult‐onset POTS, but the proportion of participants with pediatric‐onset POTS in this study was much smaller than the adult‐onset POTS group, as would be expected given that the VUMC Autonomic Dysfunction Center only sees adult patients.

The time from symptom onset to diagnosis and treatment may also play a role in long‐term outcomes, with a shorter time leading to a larger proportion of improved patients [7, 8]. In a survey of approximately 4800 patients with POTS (median age of onset 17 [13, 28] years), 42% reported that their symptoms had generally improved, 10% had no change, and 44% had worsened symptoms since their initial symptom onset [18]. This is also lower than most of the pediatric studies [7, 9, 13], and similar to our study where 46% of patients reported improvement.

Despite receiving care for POTS, symptoms persisted for decades after initial onset. Even though just under half of the group reported improvement, over 75% of survey respondents were still experiencing POTS symptoms, including brain fog, palpitation with rapid heartbeat, lightheadedness, and headaches. Brain fog is a particularly troubling symptom that causes impairment in school, work and social settings [20].

### Comorbidities

Most participants reported one or more of the common POTS comorbidities. More patients had irritable bowel syndrome, autoimmune disease, myalgic encephalomyelitis/chronic fatigue syndrome, and neuropathy than other findings [18, 21]. The proportion of patients with gastroparesis and mast cell activation syndrome (MCAS) were similar to a large survey [18]. The proportion of patients with hypermobile Ehlers–Danlos syndrome (EDS) was lower than other findings ranging from 25% to 30% [18, 22, 23]. It is possible that EDS is more commonly found in patients with a younger age of POTS onset and is therefore less prevalent in this group of patients with POTS onset at an older age [24].

Comorbidities are likely a strong factor in determining outcomes in POTS. All but one participant in NOT IMPROVED had one or more of neuropathy, gastroparesis and overactive bladder symptoms, compared to only 20% of IMPROVED. An association between neuropathy and gastrointestinal and bladder symptoms bas been recognized in patients with POTS [25]. Overactive bladder symptoms are common in patients with POTS [26], and bladder dysfunction can result from peripheral neuropathy [27]. Gastroparesis is also associated with neuropathy [28]. These findings may point toward an underlying neuropathy that leads to worsening POTS symptoms over time in this subgroup. Further research is needed to explore this association.

The reductions (improvement) in total COMPASS‐31 score, as well as the OI domain at the time of the survey, were comparable to other shorter term follow‐up studies of POTS patients [8, 12, 29]. In contrast, the pupillomotor scores increased over time. The gastrointestinal and bladder scores also increased, but these were not significantly different after Bonferroni correction. This may indicate that current POTS treatments are helpful in targeting OI symptoms but are less effective at treating GI symptoms. This may also reflect the challenges with diagnosing and treating conditions related to POTS including MCAS. Symptoms at the time of initial evaluation did not appear to predict long‐term outcomes. There were no significant differences in COMPASS‐31 at the time of evaluation, as recalled by participants. NOT IMPROVED participants reported their overall health (on a 0–100 scale) as better at the time of first VUMC visit compared to IMPROVED. In a study of POTS patients with and without neuropathy, patients with neuropathy rated their health‐related quality of life as better than those with no neuropathy [25]. It is possible that patients with neuropathy have a slower deterioration of symptoms over time, whereas POTS patients without neuropathy are initially worse, and have poorer quality of life, but improve over time. More patients in the NOT IMPROVED group reported neuropathy at the time of the survey and this could explain their higher initial scores. Thus, we were not able to identify predictors of worse outcomes at the time of initial diagnosis, highlighting the importance of clinical follow‐up in these patients.

### Medical care and treatment for POTS over time

POTS patients required ongoing care in the long term. For those who were not seeing a PCP, less than half indicated that the reason was because their POTS symptoms were well managed. There were other contributing factors. This demonstrates a requirement for continued care, and not simply that patients are better and no longer require medical intervention.

Continued medication and non‐pharmacological treatment use was required for almost all participants in the study, with over half using both pharmacological and non‐pharmacological treatments. This was not a group of patients receiving inadequate treatment. The proportion of participants, continuing to take medications for POTS, was similar to an adult POTS follow‐up study [10], but higher than in the pediatric follow‐up studies [9, 13].

### Employment, disability, and social impacts of POTS

The rates of employment were comparable to a large POTS survey [6], but the proportion unable to work due to POTS, and proportion who had applied for disability benefits, were higher than in other studies [5, 18]. The lower proportion of participants working at the time of the survey, compared to the time of diagnosis, demonstrates that even with treatment, some participants had to stop working due to POTS. This indicates a high burden of symptoms and disability in this group.

### Clinical implications

Given the diverse and persistent symptoms experienced by this patient group, a collaborative care delivery model with a long‐term clinical home for this patient group should be considered. In this model, patients would have access to continuous follow‐up, rather than sporadic care when symptoms worsen [30, 31, 32].

### Limitations

#### Long‐term engagement

One criticism of long‐term follow‐up studies is that participants who continue to have symptoms may be more likely to remain engaged in research opportunities. To help reduce this effect, participants were recruited based on past research records, and ongoing follow‐up at VUMC was not required. If current contact information was not available for specific participant, however, then they were not able to be contacted for this study. We cannot, however, rule out the possibility that the proportion of patients that improved was higher (or lower) in those lost to follow‐up or those choosing not to participate in the survey.

#### Sample size, selection, and recall bias

As a specialist autonomic center, VUMC might see a specific population of POTS patients who might have more severe symptoms, as participants were willing to travel to a specialist center. This group of adult‐onset patients may represent a cohort with a different pathophysiology than patients with a pediatric onset. This study is smaller than other larger POTS survey studies, and this could introduce bias. The small sample size may mean that some statistical analyses were underpowered and therefore unable to detect a difference. The nonresponder rate of 38% is also a limitation. Multiple attempts were made to contact individuals who initially expressed interest, but it is unknown why they chose to not complete the survey. The advantage of this patient population, however, is that they have a confirmed diagnosis of POTS and did have access to high quality care. A large POTS survey compared POTS patients seen at an expert autonomic site (such as VUMC) to those not seen at one of these sites [18], and the two groups were largely similar, suggesting that this referral bias might not have significantly skewed our results. It is still important, however, to consider how this cohort of patients may be different from patients who were not treated as a specialist autonomic center. Participants were asked to recall information from the time of symptom onset and diagnosis, which was >10 years prior. This could have introduced recall bias and should be considered as a limitation, especially with standardized scores like the COMPASS‐31.

### Future directions

Future research could further explore these findings in a larger sample. Specifically, investigation of the role of comorbid diagnoses in outcomes and differences in responses to treatments, such as exercise, may help to further understand outcomes in this patient group. Additionally, the not insignificant proportion of individuals requiring ongoing medical care for several years after onset and diagnosis demonstrates the important need for physician knowledge of POTS and an increased number of physicians with the ability to care for these patients.

### Conclusions

In a cohort of adult patients with POTS who received care at a specialist autonomic center, less than half reported their POTS symptoms as improved 10 or more years after symptom onset. Most patients with POTS continued to experience symptoms for several years after diagnosis, continued to receive medical care for POTS, and remained on pharmacological and non‐pharmacological treatments. POTS is a long‐term chronic illness leading to significant disruptions in multiple areas of life and does not spontaneously resolve in most patients.

## Author contributions

**Kate M. Bourne**: Conceptualization; investigation; writing—original draft; methodology; writing—review and editing; formal analysis. **Giris Jacob**: Writing—review and editing. **Amanda Peltier**: Writing—review and editing. **Alfredo Gamboa**: Writing–review and editing; conceptualization; supervision. **Juliette Hall**: Methodology; writing—review and editing. **Italo Biaggioni**: Writing—review and editing. **Cyndya A. Shibao**: Writing—review and editing. **André Diedrich**: Writing—review and editing. **Bonnie Black**: Conceptualization; methodology; project administration; writing—review and editing. **Luis Okamoto**: writing—original draft. **Robert S. Sheldon**: Writing—review and editing. **Satish R. Raj**: Conceptualization; funding acquisition; writing—review and editing; methodology; formal analysis; supervision.

## Conflict of interest statement

K.B., no disclosures to report. A.G., no disclosures to report. B.B., no disclosures to report. J.H., no disclosures to report. I.B., consultant for Theravance Biopharma, Amneal Pharmaceuticals, Regeneron Pharmaceutical, Takeda Pharmaceutical and Neurawell Therapuetics. C.A.S., advisor/consultant for Antag Therapeutics, Theravance Biopharma. C.A.S., partly funded by the American Heart Association and NIH, NHLBI, R01HL159203. A.D., partly supported by NIH, R01 HL142583. No other disclosures. A.P., no disclosures to report. G.J., C.M.O., CardiacSense, Medical Wearable Devices Company, HiTech, Cesarea, Israel. L.O., no disclosures to report. B.S., no disclosures to report. S.R.R., consultant to Regeneron, Argenx BV, Antag Pharma, and Lumia Health.

## Funding information

This work was supported in part by The National Center for Advacing Translational Sciences.  Award UL1 TR000445. S.R.R. receives research support from the Canadian Institutes of Health Research (CIHR; Ottawa, ON, Canada) grant MOP142426. C.A.S. received research support from NIH, NHLBI R01 HL159203‐01. A.D. was partly supported by NIH R01 HL142583.

## Supporting information




**Figure S1**: A. Age at symptom onset, diagnosis, and time of survey in NOT IMPROVED compared to IMPROVED. B. Number of doctors seen before diagnosis and diagnostic delay (time in years from symptom onset to diagnosis) in NOT IMPROVED compared to IMPROVED.
**Table S1**: Additional Results.
**Table S2**: Proportion of IMPROVED (I) vs NOT IMPROVED (NI) participants with common orthostatic symptoms and frequency of symptoms at the time of the survey.
**Table S3**: Common POTS comorbidities in IMPROVED compared to NOT IMPROVED at the time of the survey.
**Table S4**: COMPASS‐31 Autonomic Symptom Score at the time of first VUMC visit, time of survey, and the change from time of first VUMC visit to time of survey in NOT IMPROVED (NI) compared to IMPROVED (I) participants.

## Data Availability

The data that support the findings of this study are available on request from the corresponding author. The data are not publicly available due to privacy or ethical restrictions.
