# Supplementary material for: Long‐term outcomes in patients with postural orthostatic tachycardia syndrome with an average follow‐up of over 20 years
Source: J Intern Med. 2026 May 19;300(3):299–311. doi: 10.1111/joim.70104 (PMC13429004; doi:10.1111/joim.70104)
Supplement: Supplementary file 1 — Figure S1: A. Age at symptom onset, diagnosis, and time of survey in NOT IMPROVED compared to IMPROVED. B. Number of doctors seen before diagnosis and diagnostic delay (time in years from symptom onset to diagnosis) in NOT IMPROVED compared to IMPROVED. Table S1: Additional Results. Table S2: Proportion of IMPROVED (I) vs NOT IMPROVED (NI) participants with common orthostatic symptoms and frequency of symptoms at the time of the survey. Table S3: Common POTS comorbidities in IMPROVED compared to NOT IMPROVED at the time of the survey. Table S4: COMPASS‐31 Autonomic Symptom Score at the time of first VUMC visit, time of survey, and the change from time of first VUMC visit to time of survey in NOT IMPROVED (NI) compared to IMPROVED (I) participants. [file JOIM-300-299-s001.docx]

**Figure S1.**

**
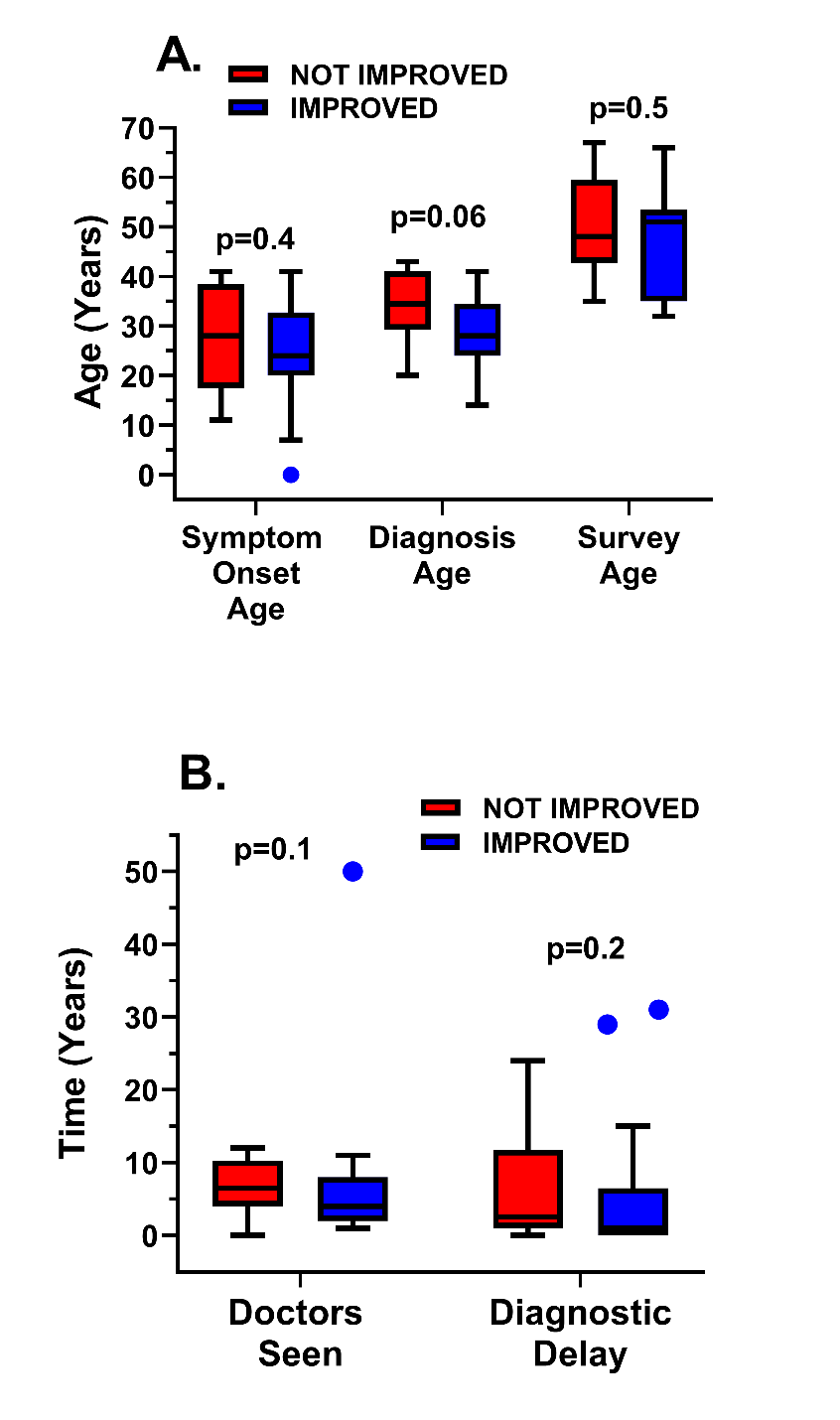
**

**Figure S1. A. Age at symptom onset, diagnosis, and time of survey in NOT IMPROVED compared to IMPROVED. B. Number of doctors seen before diagnosis and diagnostic delay (time in years from symptom onset to diagnosis) in NOT IMPROVED compared to IMPROVED.**

**Table S1. Additional Results**

***Physicians:*** Participants who were currently seeing a physician most reported that the main doctor following them for POTS was a cardiologist (36%), followed by PCP (28%), neurologist (24%), and other type of physician (12%). Of participants who were seeing a PCP for POTS at the time of the survey (40%), the median number of visits was 3 (2, 3) per year, and the median number of visits for a specialist for POTS was 2 (1, 4) per year.

***Female Participants:*** About half (59%) of post-menopausal participants reported that their menopause was surgically induced. For post-menopausal participants, the time since last menstrual period was 14 [6, 28] years. After menopause, 35% of participants reported their POTS symptoms stayed the same, 35% reported their POTS symptoms worsened, 18% reported their symptoms improved, and 12% were unsure. Almost one-quarter (23%) of participants have been pregnant since POTS diagnosis, with 30% reporting improved POTS symptoms, 30% reporting worsened symptoms, and 40% reporting variable symptoms during pregnancy.

***Impacts of POTS:*** Most participants indicated that POTS negatively impacted their marriage (57%), family relationships (57%), employment (73%), experiences in social settings (80%), friendships (64%), travel (84%), personal finances (68%), and hobbies (77%). Over half (57%) of participants have had to stop driving due to POTS symptoms at some point in time.

***Employment:*** Only 2 participants were not employed at both the time of diagnosis and the time of the survey. Participants who were employed at the time of the survey had fewer comorbidities (out of those listed in Table 2), compared to those who were unemployed (2 [1, 3] vs. 5 [3, 6], p<0.001). At the time of diagnosis, a similar proportion of NOT IMPROVED and IMPROVED were employed (75% vs. 71%, p=0.7). At the time of the survey, only a minority of NOT IMPROVED were employed (19%), compared to most of the IMPROVED participants (86%, p<0.001). Of NOT IMPROVED who were not working (n=13), 85% were unable to work due to POTS.

***Education:*** In total, 41% of participants developed POTS before completing their education, and 61% of these participants indicated POTS changed the long-term trajectory of their educational plan.

***Disability Benefits:*** Overall, 63% had applied for at least one disability benefit, with 56% applying for government disability benefits and 37% applying for benefits through a private company. Most (83%) were successful in receiving government disability benefits, though most participants (80%) required an appeal process. Similarly, 90% of participants were successful in receiving private benefits (with 33% requiring an appeal process).

**Table S2. Proportion of IMPROVED (I) vs NOT IMPROVED (NI) participants with common orthostatic symptoms and frequency of symptoms at the time of the survey.**

| **Symptom** | **HAS SYMPTOM** | | | **EVERY DAY or MOST DAYS** | | |
| --- | --- | --- | --- | --- | --- | --- |
|  | **NI**  **n (%)** | **I**  **n (%)** | **p-value** | **NI**  **n (%)** | **I**  **n (%)** | **p-value** |
| Lightheadedness | 14 (88) | 13 (62) | 0.9 | 9 (64) | 2 (15) | **0.2** |
| Tremulousness | 13 (81) | 5 (25) | **0.02** | 7 (54) | 0 (0) | 0.9 |
| Rapid Heart Beat | 15 (94) | 16 (76) | 1.0 | 13 (87) | 8 (50) | 0.5 |
| Headache | 15 (94) | 13 (62) | 0.5 | 8 (53) | 0 (0) | **0.02** |
| Nausea | 15 (94) | 6 (29) | **0.009** | 8 (53) | 0 (0) | 0.4 |
| Blurry Vision | 15 (94) | 8 (38) | **0.009** | 7 (47) | 1 (5) | 1.0 |
| Brain Fog | 15 (94) | 16 (76) | 1.0 | 12 (80) | 7 (44) | 0.6 |
| Chest Pain | 12 (75) | 9 (43) | 0.9 | 2 (17) | 0 (0) | 1.0 |
| Shortness of Breath | 13 (81) | 5 (24) | **0.009** | 9 (69) | 1 (20) | 0.9 |

NI: NOT IMPROVED. I: IMPROVED. P-values have been corrected for multiple comparisons using Bonferroni correction.

**Table S3. Common POTS comorbidities in IMPROVED compared to NOT IMPROVED at the time of the survey.**

| Comorbidity | NOT IMPROVED  n (%) | IMPROVED  n (%) | p-value |
| --- | --- | --- | --- |
| Headache | 13 (81) | 11 (52) | 0.8 |
| Irritable Bowel Syndrome | 11 (69) | 7 (35) | 0.8 |
| Neuropathy | 10 (67) | 2 (10) | **0.01** |
| Chronic Fatigue Syndrome | 7 (44) | 4 (19) | 1.0 |
| Overactive bladder symptoms | 8 (50) | 3 (14) | 0.3 |
| Autoimmune disease | 6 (38) | 3 (14) | 1.0 |
| Gastroparesis | 7 (44) | 0 (0) | **0.01** |
| Hypermobile Ehlers Danlos Syndrome | 4 (25) | 3 (14) | 1.0 |
| Mast Cell Activation Syndrome | 3 (19) | 1 (5) | 1.0 |
| Chiari Malformation | 2 (13) | 1 (5) | 1.0 |

P-values have been corrected for multiple comparisons using Bonferroni correction.

**Table S4. COMPASS-31 Autonomic Symptom Score at the time of first VUMC visit, time of survey, and the change from time of first VUMC visit to time of survey in NOT IMPROVED (NI) compared to IMPROVED (I) participants.**

| **Domain** | **Time of First VUMC Visit** | | | **Time of Survey** | | | **Change**  **(Survey –Initial VUMC Visit)** | | |
| --- | --- | --- | --- | --- | --- | --- | --- | --- | --- |
|  | **NI** | **I** | **p-value** | **NI** | **I** | **p-value** | **NI** | **I** | **p-value** |
| Orthostatic Intolerance | 22 (20, 27) | 24 (22, 28) | 0.7 | 20 (20, 24) | 12 (8, 16) | **0.007** | -2 (-7, 0) | -12 (-16, -8) | **0.007** |
| Vasomotor | 2 (2, 3) | 2 (2, 3) | 1.0 | 3 (3, 3) | 2 (2, 3) | 0.1 | 0.8 (0, 2) | 0 (-1, 0) | 0.06 |
| Secretomotor | 9 (9 9) | 9 (9, 9) | 1.0 | 10 (9, 11) | 9 (9, 11) | 1.0 | 0 (0, 2) | 0 (-2, 2) | 1.0 |
| Gastrointestinal | 8 (5, 11) | 7 (5, 10) | 1.0 | 10 (9, 14) | 8 (6, 11) | 0.2 | 2 (-1, 4) | 0 (-2, 2) | 1.0 |
| Bladder | 0 (0, 2) | 0 (0, 1) | 1.0 | 2 (1, 3) | 0 (0, 2) | 0.1 | 1 (-1, 3) | 0 (0, 1) | 0.3 |
| Pupillomotor | 2 (0, 2) | 1 (0, 2) | 1.0 | 3 (2, 3) | 2 (1, 2) | 0.2 | 2 (1, 2) | 0 (0, 2) | 0.5 |
| **Total** | 44 (37, 51) | 47 (41, 52) | 1.0 | 49 (40, 52) | 36 (26, 43) | **0.007** | 2 (-2, 8) | -11 (-16, -9) | **0.007** |

NI: NOT IMPROVED. I: IMPROVED. P-values have been corrected for multiple comparisons using Bonferroni correction.

**Custom Survey Questions:**

**Demographics:**

What is your age? _____

What is your biological sex (at birth)?

- Male
- Female
- Prefer not to say

What is your Gender Identity?

- Cis-Woman (a woman since birth)
- Cis-Man (a man since birth)
- Trans-Man (Woman to Man)
- Trans-Woman (Man to Woman)
- Non-binary
- Prefer to self-describe
- Prefer not to say

Please self-describe your gender identity: ____________________

What is your Race?

- White
- Black
- East Asian
- South Asian
- Hawaiian or Pacific Islander
- Native or Indigenous
- Multiple
- Other
- Prefer not to say

What is your ethnicity?

- Hispanic
- Non-Hispanic
- Prefer not to say

**Past POTS:**

What year did your POTS symptoms start: ___________________

What year were you diagnosed with POTS:___________________

If known, how many different doctors did you see before your diagnosis at Vanderbilt (VUMC)? _______

What year were you first seen at Vanderbilt (VUMC)? ______

**Symptoms:**

Have you continued to have POTS symptoms since diagnosis/last seen at VUMC?

- Yes
- No

Overall, since diagnosis, have your symptoms:

- Completely resolved
- Improved
- Worsened
- Variable
- No Change
- Unsure
- Prefer not to say

Shortly after diagnosis (under 1 year) did your symptoms:

- Completely resolved
- Improved
- Worsened
- Variable
- No Change
- Unsure
- Prefer not to say

5 years after diagnosis, did your symptoms:

- Completely resolved
- Improved
- Worsened
- Variable
- No Change
- Unsure
- Prefer not to say

How would you rate your POTS symptoms now, compared to your normal health before your POTS symptoms began?

- Completely resolved
- Improved
- Worsened
- Variable
- No Change
- Unsure
- Prefer not to say

**Do you currently experience any of the following symptoms?**

Lightheadedness

- Yes
- No
- Prefer not to say

How often do you experience lightheadedness?

- Everyday
- Most days (e.g. 4 of the 7 days of the week or more)
- Some days (e.g. 1 or 2 times a week)
- Rarely (less than once a week)

Tremulousness

- Yes
- No
- Prefer not to say

How often do you experience tremulousness?

- Everyday
- Most days (e.g. 4 of the 7 days of the week or more)
- Some days (e.g. 1 or 2 times a week)
- Rarely (less than once a week)

Rapid Heart Beat

- Yes
- No
- Prefer not to say

How often do you experience rapid heart beat?

- Everyday
- Most days (e.g. 4 of the 7 days of the week or more)
- Some days (e.g. 1 or 2 times a week)
- Rarely (less than once a week)

Headache

- Yes
- No
- Prefer not to say

How often do you experience headache?

- Everyday
- Most days (e.g. 4 of the 7 days of the week or more)
- Some days (e.g. 1 or 2 times a week)
- Rarely (less than once a week)

Nausea

- Yes
- No
- Prefer not to say

How often do you experience nausea?

- Everyday
- Most days (e.g. 4 of the 7 days of the week or more)
- Some days (e.g. 1 or 2 times a week)
- Rarely (less than once a week)

Blurry Vision

- Yes
- No
- Prefer not to say

How often do you experience blurry vision?

- Everyday
- Most days (e.g. 4 of the 7 days of the week or more)
- Some days (e.g. 1 or 2 times a week)
- Rarely (less than once a week)

Brain Fog

- Yes
- No
- Prefer not to say

How often do you experience brain fog?

- Everyday
- Most days (e.g. 4 of the 7 days of the week or more)
- Some days (e.g. 1 or 2 times a week)
- Rarely (less than once a week)

Chest Pain

- Yes
- No
- Prefer not to say

How often do you experience chest pain?

- Everyday
- Most days (e.g. 4 of the 7 days of the week or more)
- Some days (e.g. 1 or 2 times a week)
- Rarely (less than once a week)

Shortness of breath

- Yes
- No
- Prefer not to say

How often do you experience shortness of breath?

- Everyday
- Most days (e.g. 4 of the 7 days of the week or more)
- Some days (e.g. 1 or 2 times a week)
- Rarely (less than once a week)

**General Health:**

Other than POTS, have you been generally well?

- Yes
- No
- Prefer not to say

After your POTS diagnosis, and after attending VUMC, have you experienced an event that caused a worsening of your POTS symptoms? (e.g. head injury, motor vehicle accident)?

- Yes
- No
- Prefer not to say

Please explain: ________________________

Have your worsening POTS symptoms resolved since?

- Yes
- No
- Prefer not to say

**Comorbidities:**

**Are you diagnosed with any of the following conditions?**

Ehlers-Danlos Syndrome:

- Yes
- No
- Prefer not to say

What type of EDS?

- Classical
- Hypermobile
- Vascular
- Other

Hypermobility Syndrome/Hypermobility Spectrum Disorder

- Yes
- No
- Prefer not to say

Mast Cell Activation Syndrome

- Yes
- No
- Prefer not to say

Chronic Fatigue Syndrome

- Yes
- No
- Prefer not to say

Gastroparesis

- Yes
- No
- Prefer not to say

Headache

- Yes
- No
- Prefer not to say

Neuropathy:

- Yes
- No
- Prefer not to say

Irritable Bowel Syndrome:

- Yes
- No
- Prefer not to say

Overactive Bladder Syndrome:

- Yes
- No
- Prefer not to say

Chiari Malformation:

- Yes
- No
- Prefer not to say

Autoimmune Disorder:

- Yes
- No
- Prefer not to say

**Current Medication:**

**Do you take, or have you taken, any of the following medication for POTS?**

Beta-blocker (e.g. propranolol, metoprolol, bisoprolol or any other medication ending in -olol)

- Yes, currently taking
- Have taken
- No

Please specify which beta-blockers (if known): ____________________

How do/did Beta-Blocker affect your POTS symptoms?

- Improved a lot
- Improved a little
- No change
- Worsened a little
- Worsened a lot
- Unsure
- Prefer not to say

Midodrine (proamatine)

- Yes, currently taking
- Have taken
- No

How does/did Midodrine affect your POTS symptoms?

- Improved a lot
- Improved a little
- No change
- Worsened a little
- Worsened a lot
- Unsure
- Prefer not to say

Fludrocortisone (Florinef)

- Yes, currently taking
- Have taken
- No

How does/did fludrocortisone affect your POTS symptoms?

- Improved a lot
- Improved a little
- No change
- Worsened a little
- Worsened a lot
- Unsure
- Prefer not to say

Modafinil (Alertec or Provigil)

- Yes, currently taking
- Have taken
- No

How does/did Modafinil affect your POTS symptoms?

- Improved a lot
- Improved a little
- No change
- Worsened a little
- Worsened a lot
- Unsure
- Prefer not to say

Armodafinil (Nuvigil)

- Yes, currently taking
- Have taken
- No

How does/did Armodafinil affect your POTS symptoms?

- Improved a lot
- Improved a little
- No change
- Worsened a little
- Worsened a lot
- Unsure
- Prefer not to say

Ivabradine (Corlanor)

- Yes, currently taking
- Have taken
- No

How does/did Ivabradine affect your POTS symptoms?

- Improved a lot
- Improved a little
- No change
- Worsened a little
- Worsened a lot
- Unsure
- Prefer not to say

Pyridostigmine (Mestinon)

- Yes, currently taking
- Have taken
- No

How does/did Pyridostigmine affect your POTS symptoms?

- Improved a lot
- Improved a little
- No change
- Worsened a little
- Worsened a lot
- Unsure
- Prefer not to say

Desmopressin (DDAVP)

- Yes, currently taking
- Have taken
- No

How does/did Desmopressin affect your POTS symptoms?

- Improved a lot
- Improved a little
- No change
- Worsened a little
- Worsened a lot
- Unsure
- Prefer not to say

Methyl-dopa (Alpha-methyldopa, aldomet)

- Yes, currently taking
- Have taken
- No

How does/did Methyl-dopa affect your POTS symptoms?

- Improved a lot
- Improved a little
- No change
- Worsened a little
- Worsened a lot
- Unsure
- Prefer not to say

Clonidine (Catapres)

- Yes, currently taking
- Have taken
- No

How does/did Clonidine affect your POTS symptoms?

- Improved a lot
- Improved a little
- No change
- Worsened a little
- Worsened a lot
- Unsure
- Prefer not to say

Low Dose Naltrexone (ReVia, Vivitrol)

- Yes, currently taking
- Have taken
- No

How does/did Low Dose Naltrexone affect your POTS symptoms?

- Improved a lot
- Improved a little
- No change
- Worsened a little
- Worsened a lot
- Unsure
- Prefer not to say

Other POTS medications

- Yes, currently taking
- Have taken
- No

Please specify which other medications you are taking for POTS: _____________

Please specify which other medications you have taken in the past for POTS: _________

How did these medications affect your POTS symptoms:

- Improved a lot
- Improved a little
- No change
- Worsened a little
- Worsened a lot
- Unsure
- Prefer not to say

**Non-Pharmacological treatments**

**Do you use any of the following non-medication treatments for POTS**

Increased salt in your diet

- Yes, currently taking
- Have taken
- No

How did increased salt affect your POTS symptoms?

- Improved a lot
- Improved a little
- No change
- Worsened a little
- Worsened a lot
- Unsure
- Prefer not to say

Increased fluid in your diet

- Yes, currently taking
- Have taken
- No

How did increased fluid affect your POTS symptoms?

- Improved a lot
- Improved a little
- No change
- Worsened a little
- Worsened a lot
- Unsure
- Prefer not to say

Electrolyte Supplements

- Yes, currently taking
- Have taken
- No

How did electrolyte supplements affect your POTS symptoms?

- Improved a lot
- Improved a little
- No change
- Worsened a little
- Worsened a lot
- Unsure
- Prefer not to say

Compression Garments

- Yes, currently taking
- Have taken
- No

How did compression garments affect your POTS symptoms?

- Improved a lot
- Improved a little
- No change
- Worsened a little
- Worsened a lot
- Unsure
- Prefer not to say

IV Saline (Emergency use only e.g. a few times per year or less)

- Yes, currently taking
- Have taken
- No

How did IV Saline (Emergency only) affect your POTS symptoms?

- Improved a lot
- Improved a little
- No change
- Worsened a little
- Worsened a lot
- Unsure
- Prefer not to say

Exercise Program

- Yes, currently taking
- Have taken
- No

How did an exercise program affect your POTS symptoms?

- Improved a lot
- Improved a little
- No change
- Worsened a little
- Worsened a lot
- Unsure
- Prefer not to say

IVIG

- Yes, currently taking
- Have taken
- No

How did IVIG affect your POTS symptoms?

- Improved a lot
- Improved a little
- No change
- Worsened a little
- Worsened a lot
- Unsure
- Prefer not to say

EPO (Epogen, Procrit)

- Yes, currently taking
- Have taken
- No

How did EPO affect your POTS symptoms?

- Improved a lot
- Improved a little
- No change
- Worsened a little

**Female Patients Only:**

What is your gynecological status?

- Pre-menopausal
- Post-menopausal
- Prefer not to say

Start date of last menstrual period: ________________

Was your menopause:

- Natural
- Medically induced
- Surgically induced

What happened to your POTS symptoms overall after menopause?

- Improved
- Stayed the same
- Worsened
- Unsure
- Variable
- Prefer not to say

Since POTS diagnosis, have you been through a pregnancy and been through the birth of a child?

- Yes
- No
- Prefer not to say

How did you symptoms change during pregnancy?

- Improved
- Stayed the same
- Worsened
- Unsure
- Variable
- Prefer not to say

After delivery, and in the short term (up to 3 months after delivery), what happened to your POTS symptoms?

- Returned to how they were before I was pregnant
- Stayed the same as when I was pregnant
- Got worse than before I was pregnant
- Unsure

After delivery, and in the long term, what happened to your POTS symptoms?

- Returned to how they were before I was pregnant
- Stayed the same as when I was pregnant
- Got worse than before I was pregnant
- Got better then before I was pregnant
- Unsure

**Social, Educational and Employment Impacts of POTS**

**Education**

Did you develop POTS before completing your education?

- Yes
- No
- Prefer not to say

In the long term, did POTS change the trajectory of your educational plan?

- Yes
- No
- Prefer not to say

**Employment**

Were you employed outside the home at the time of POTS diagnosis?

- Yes
- No
- Prefer not to say

Select:

- Full-time employment
- Part-time employment

Why were you working part time?

- I was unable to work outside the home full time due to POTS
- I was unable to work outside the home full time due to another medical condition
- I was not working outside the home full time for another reason unrelated to my health
- Prefer not to say

Are you currently employed outside the home?

- Yes
- No
- Prefer not to say

Is your current employment outside the home:

- Full Time
- Part Time
- Prefer not to say

Why are you currently unemployed outside the home?

- I am unable to work outside the home due to POTS symptoms
- I am unable to work outside the home due to a medical condition other than POTS
- I am unable to work outside the home for another reason unrelated to my health
- I am retired
- Prefer not to say

In the long term, did POTS change your career?

- Yes
- No
- Prefer not to say

**Social Impacts:**

Has having POTS impacted any of the following?

Marriage

- Yes
- No
- Prefer not to say

Family Relationships

- Yes
- No
- Prefer not to say

Employment

- Yes
- No
- Prefer not to say

Social Settings

- Yes
- No
- Prefer not to say

Friendships

- Yes
- No
- Prefer not to say

Travel

- Yes
- No
- Prefer not to say

Personal Finances

- Yes
- No
- Prefer not to say

Hobbies

- Yes
- No
- Prefer not to say

Please explain how POTS impacted your marriage: _______________

Please explain how POTS impacted your family relationships: _______________

Please explain how POTS impacted your employment: _______________

Please explain how POTS impacted your social settings: _______________

Please explain how POTS impacted your friendships: _______________

Please explain how POTS impacted your travel: _______________

Please explain how POTS impacted your personal finances: _______________

Please explain how POTS impacted your hobbies: _______________

Have you ever had to stop driving due to POTS symptoms?

- Yes
- No
- Prefer not to say

**Disability Benefits**

Have you every applied for disability benefits?

- Yes
- No
- Prefer not to say

Did you apply for disability benefits through a private insurance company?

- Yes
- No
- Prefer not to say

Was your application successful?

- Yes
- No
- Prefer not to say

Did you receive your private insurance benefits through an appeals process?

- Yes
- No
- Prefer not to say

Did you apply for government disability benefits (SSD)?

- Yes
- No
- Prefer not to say

Was your application successful?

- Yes
- No
- Prefer not to say

Did you receive your government benefits through an appeal process?

- Yes
- No
- Prefer not to say

**Physicians and Procedures:**

Do you currently see your primary care physician for POTS symptoms on a regular basis?

- Yes
- No
- Prefer not to say

How many times per year? _______________

Why not? (select all that apply)

- My symptoms are well managed/not needed
- Doctor is too far away from me/where I live
- Can’t find a doctor who can help with POTS
- Insurance/Financial issue
- Other (please specify)

Specification: _________________________

Are you currently being followed by a specialist physician for POTS?

- Yes
- No
- Prefer not to say

Please select which specialty/specialties apply:

Cardiologist

- Yes
- No

Neurologist

- Yes
- No

Gastroenterologist

- Yes
- No

Other (please specify)

Specification: _____________________

What specialty is the main doctor who follows you for POTS:

- Primary care physician
- Cardiologist/electrophysiologist
- Neurologist
- Gastroenterologist
- Other
- Prefer not to say

How many times per year do you see the main specialist who follows you for POTS symptoms? ________

In total how many different specialists or doctors do you see for POTS and related conditions (e.g. EDS, MCAS)? _____________
